# Chromosome-Level Genome Assembly of the Viviparous Eelpout *Zoarces viviparus*

**DOI:** 10.1093/gbe/evae155

**Published:** 2024-07-17

**Authors:** Nico Fuhrmann, Marie V Brasseur, Christina E Bakowski, Lars Podsiadlowski, Stefan Prost, Henrik Krehenwinkel, Christoph Mayer

**Affiliations:** Department of Biogeography, Trier University, Trier 54296, Germany; Department of Biogeography, Trier University, Trier 54296, Germany; Centre for Molecular Biodiversity Research, Leibniz Institute for the Analysis of Biodiversity Change (LIB), Bonn 53113, Germany; Centre for Molecular Biodiversity Research, Leibniz Institute for the Analysis of Biodiversity Change (LIB), Bonn 53113, Germany; Ecology and Genetics Research Unit, University of Oulu, Oulu 90014, Finland; South African National Biodiversity Institute, National Zoological Garden, Pretoria 0002, South Africa; Central Research Laboratories, Natural History Museum Vienna, Vienna 1010, Austria; Department of Biogeography, Trier University, Trier 54296, Germany; Centre for Molecular Biodiversity Research, Leibniz Institute for the Analysis of Biodiversity Change (LIB), Bonn 53113, Germany

**Keywords:** Zoarcidae, reference genome, bioindicator, marine environmental pollution, viviparous blenny

## Abstract

The viviparous eelpout *Zoarces viviparus* is a common fish across the North Atlantic and has successfully colonized habitats across environmental gradients. Due to its wide distribution and predictable phenotypic responses to pollution, *Z. viviparus* is used as an ideal marine bioindicator organism and has been routinely sampled over decades by several countries to monitor marine environmental health. Additionally, this species is a promising model to study adaptive processes related to environmental change, specifically global warming. Here, we report the chromosome-level genome assembly of *Z*. *viviparus*, which has a size of 663 Mb and consists of 607 scaffolds (N50 = 26 Mb). The 24 largest represent the 24 chromosomes of the haploid *Z. viviparus* genome, which harbors 98% of the complete Benchmarking Universal Single-Copy Orthologues defined for ray-finned fish, indicating that the assembly is highly contiguous and complete. Comparative analyses between the *Z*. *viviparus* assembly and the chromosome-level genomes of two other eelpout species revealed a high synteny, but also an accumulation of repetitive elements in the *Z*. *viviparus* genome. Our reference genome will be an important resource enabling future in-depth genomic analyses of the effects of environmental change on this important bioindicator species.

**Significance**

*Zoarces viviparus* is a bioindicator species of international significance and a highly promising model system to study the mechanisms underlying the differential thermal tolerance of marine ectotherms. In this study, we present a chromosome-level reference genome of *Z*. *viviparus*, an essential resource to study the genomic imprints of habitat pollution, ocean warming, and other environmental changes in marine fish.

## Introduction

**Fig. 1.**
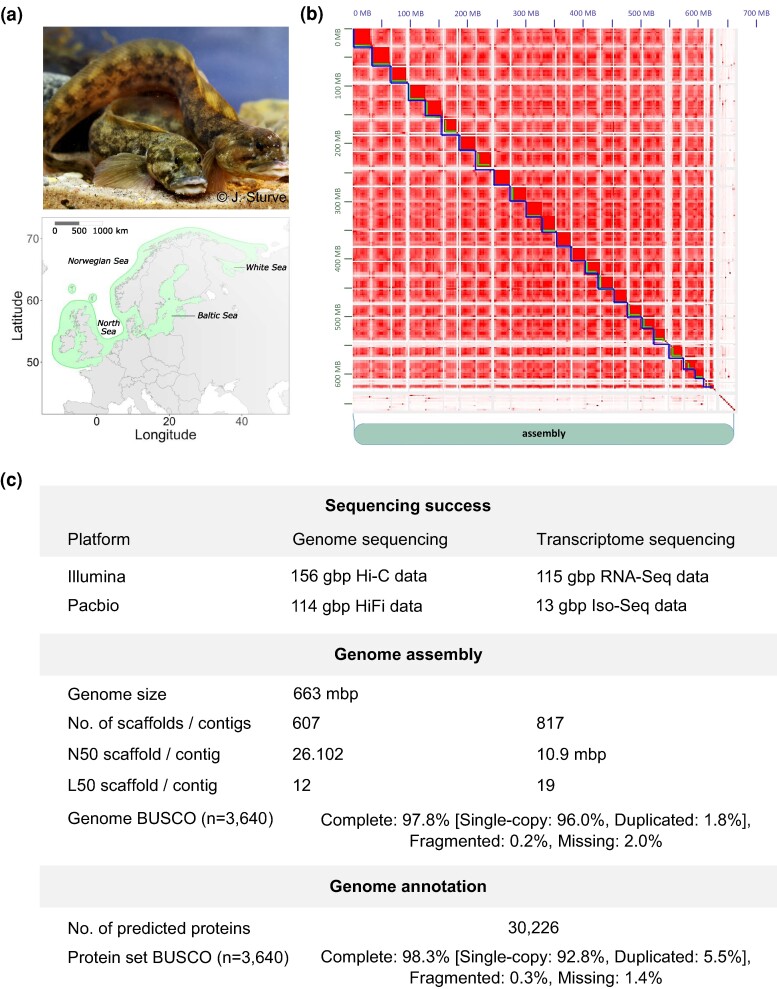
*Zoarces viviparus* and its distribution area in the North Atlantic (a), Hi-C contact density map of the *Z*. *viviparus* assembly indicating 24 main scaffolds that represent the 24 chromosomes of the haploid *Z*. *viviparus* genome (b) and sequencing success and genome assembly statistics (c). The photograph of the *Z. viviparus* specimens was kindly provided by Joachim Sturve.

The viviparous eelpout *Zoarces viviparus* L. is a common fish species in the North Atlantic. Its wide latitudinal distribution area ranges from the English Channel to the northern coast of Norway, including the brackish water habitats of the Baltic Sea (Fig. 1a). As the species is philopatric throughout its life, local populations are exposed to differential habitat conditions related to both natural factors such as temperature and salinity gradients and anthropogenic activities, e.g. specific types of pollution. Given its sensitivity to environmental pollution, which results in distinct physiological responses and phenotypic alterations, its philopatric lifestyle, and its wide distribution, *Z*. *viviparus* is used as an ideal marine bioindicator organism (Jacobsson et al. 1986; Hedman et al. 2011). Moreover, its viviparous mode of reproduction allows assessing the effect of contaminants directly on sensitive life stages, i.e. larvae, and quantifying the reproductive success of female individuals (e.g. Bergek et al. 2012; Brande-Lavridsen et al. 2013). For these reasons, *Z*. *viviparus* has been extensively used in ecotoxicological studies (Hedman et al. 2011) and is the selected sentinel species in national and international marine monitoring projects (OSPAR, https://www.ospar.org; HELCOM, https://helcom.fi). Environmental specimen banks (ESBs) have sampled and stored tissue from *Z*. *viviparus* populations over decades (Klein et al. 2018). These samples are cryo-conserved in liquid nitrogen, ensuring an excellent preservation of DNA and RNA (Krehenwinkel et al. 2022), and provide the invaluable opportunity to study the molecular signatures of habitat degradation in the North Atlantic through time and space. To exploit the full potential of present and future samples, a high-quality reference genome of *Z*. *viviparus,* serving as a backbone for genomic and transcriptomic analyses, is essential.

Such a reference genome additionally constitutes an important resource for molecular ecologists and evolutionary biologists studying the genomic mechanisms involved in acclimatization and adaptation to changing environments, specifically global warming. Although being considered cold-adapted, *Z*. *viviparus* has colonized habitats across a large temperature gradient. Therefore, this species has been used as a model system to show that the thermal limit of marine ectotherms is mainly driven by temperature-limited oxygen supply (Pörtner and Knust 2007). This supports previous studies reporting an elevated hypoxia tolerance of *Z*. *viviparus* (Fischer et al. 1992; Zakhartsev et al. 2003), which might have contributed to its ability to cope better with heat stress than, for example, its stenothermic relative *Pachycara brachycephalum* (Van Dijk et al. 1999). As eelpouts (Zoarcidae) are found in various types of marine habitats and have a cosmopolitan distribution, they are ideally suited for comparative genomic studies focusing on adaptive processes. Yet, only three eelpout genomes are available at the National Center for Biotechnology Information (NCBI) genome database, and only two are chromosome-level genomes (NCBI Genome database, accessed 2024 April 3). In this study, we contribute to closing this resource gap and present the first, high-quality chromosome-level reference genome of *Z*. *viviparus*, a bioindicator species of international significance.

## Results and Discussion

### Genome Sequencing and Assembly Metrics

DNA sequencing produced 114 giga base pairs (gbp) PacBio HiFi data (N50 = 12,396 bp) and 156 gbp Illumina Hi-C data (Fig. 1c). Using the average assembly coverage of 164.04 ± 75.71 (mean ± SD) derived from long-read alignments, we calculated an estimated genome size of approximately 694 mega base pairs (mbp). Our final genome assembly consists of 663 mbp, which is similar to the assembly sizes of the other chromosome-level eelpout genomes from *Melanostigma gelatinosum* (662 mbp) (Bista et al. 2024) and *Lycodes pacificus* (646.4 mbp). We assembled 607 scaffolds (N50 = 26 mbp), whereby 94% of all bases are present in the 24 largest scaffolds (Fig. 1b and supplementary figs. S1a and S2, Supplementary Material online). These likely represent the 24 chromosomes of the haploid *Z*. *viviparus* genome previously identified in karyograms (Yershov 2005). The GC content among the scaffolds is consistent, and no evidence for contamination was found (supplementary figs. S1b and S2, Supplementary Material online). From the searched Benchmarking Universal Single-Copy Orthologues (BUSCOs) (Manni et al. 2021) defined for ray-finned fish (Actinopterygii_odb10), 97.8% were identified as being complete (Fig. 1c and supplementary fig. S2, Supplementary Material online). Additionally, we assembled the mitochondrial genome with a total size of 16,833 bp and annotated its 13 protein-coding genes, 22 tRNAs, 2 rRNAs, and the D-loop control region (supplementary fig. S4, Supplementary Material online).

### Transcriptome Sequencing and Genome Annotation

Illumina RNA sequencing (RNA-seq) produced 583,912,676 reads (115 gbp), from which 574,213,064 reads were retained after quality trimming. Of these, 96.2% ± 0.3 per library aligned to the assembled genome. PacBio Iso-Seq yielded 3,634,738 reads (7 gbp) and 4,334,131 reads (6 gbp) from the muscle (N50 = 1.8 kilo base pairs) and the liver (N50 = 1.7 kilo base pairs) transcriptomes, respectively, of which >99% aligned to the genome. This mapping information was used to predict a set of 30,226 proteins, which includes 98.3% complete BUSCO protein models.

### Comparative Analysis

**Fig. 2.**
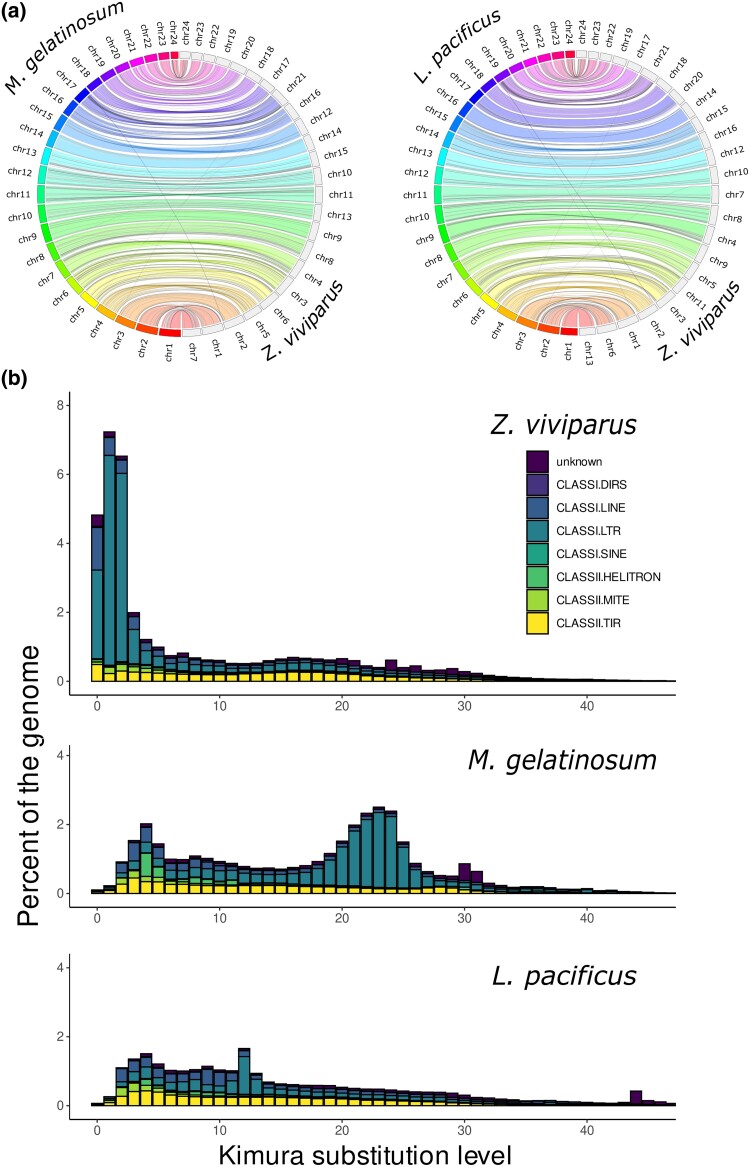
A comparison of the *Z*. *viviparus* genome with the two other chromosome-level genome assemblies of eelpouts present in the NCBI. While the synteny is highly conserved between the eelpout genomes (a), a recent expansion of LTRs was observed in the repeat landscape of *Z*. *viviparus* (b).

Although the genomic architecture in fish is highly diverse, the synteny in Actinopterygii genomes is well-conserved (Stemshorn et al. 2005; Nolte 2020) and no major structural rearrangements between the *Z*. *viviparus* genome and the two other eelpout genomes were observed (Fig. 2a). Interestingly, their genomes show distinct repeat landscapes: the *Z*. *viviparus* genome harbors many long tandem repeats (TRs), the longest being a compound repeat with a total length of 1,831,213 bp. The 29 TRs with a length of >100,000 bp are based only on a small number of repeat units (supplementary table S1, Supplementary Material online). While the TR content is comparable between the genomes of *Z. viviparus* and *L. pacificus* (∼7%), the genome of *M. gelatinosum* shows a relatively high TR content of >10% (supplementary figs. S5 and S6, Supplementary Material online). At the same time, we found evidence for a relatively recent expansion of long terminal repeat (LTR) elements in *Z. viviparus*, accounting for almost 13% of its genome, as compared to <2% in the two other species (Fig. 2b). While transposon bursts may disturb gene regulation by elements being copied into introns or regulatory elements adjacent to the genes, these events can also provide an opportunity for rapid adaptation to environmental change by, for example, enhancing a population's diversity in gene expression (Casacuberta and González 2013).

## Conclusion

We present a high-quality genome assembly for the viviparous eelpout *Z*. *viviparus*, an important marine bioindicator organism. This chromosome-level reference genome paves the way for future research focusing on the molecular mechanisms shaping the acclimatization and adaptation of *Z*. *viviparus* to environmental change.

## Materials and Methods

### Specimen Sampling, DNA Extraction and Sequencing

The specimen used for DNA extraction was caught by fisherman in May 2022 in the Meldorf Bay, Germany (location: latitude 54.10, longitude 8.80), transported to the German ESB, and killed according to German animal protection laws (for details, see Klein et al. 2018). Muscle and liver tissues of the specimen were dissected and preserved in liquid nitrogen for long-term storage at the ESB.

For long-read sequencing, DNA was extracted from ∼20 mg of liver tissue using the Monarch HMW DNA extraction kit (New England Biolabs). A 1.5% agarose gel was used to check whether the DNA was successfully extracted. For further library preparation and sequencing, the extracted DNA was sent to the West German Genome Center (WGGC) in Düsseldorf, Germany. Library preparation was performed using the SMRTbell prep kit 3.0 (PacBio), and the sample was sequenced on a PacBio Sequel II device using the HiFi mode.

For chromatin conformation capture, DNA was extracted from ∼100 mg of muscle tissue and a Hi-C proximity ligation library was prepared using the Arima Hi-C+ kit (Arima Genomics). The library was indexed with the Accel-NGS 2S Plus DNA library kit (Swift Biosciences) and sent to the WGGC for paired-end sequencing (150 bp) on an Illumina NextSeq 2000.

### Mitochondrial Genome Assembly and Annotation

The Vulcan pipeline v.1.0.3 (Fu et al. 2021) was used to extract mitochondrial reads in the PacBio HiFi data. First, mitochondrial reads were identified by aligning all reads to the mitogenome of *Pholis gunnellus* (GenBank NC_052755, v1). These reads were fastq-transformed with seqtk v.1.4-r122 (Li 2024, https://github.com/lh3/seqtk) and assembled using Flye v.2.9.2-b1786 (Kolmogorov et al. 2019) with an expected genome size of 17 kilo base pairs and an asm-coverage of 50. The mitogenome annotation was performed with MitoAnnotator v.3.92 (Iwasaki et al. 2013; Sato et al. 2018; Zhu et al. 2023).

### Genome Assembly and Repetitive Sequence Content

PacBio HiFi and Illumina Hi-C reads were assembled with Hifiasm v.0.19.4-r575 (Cheng et al. 2021). Assembly scaffolding was performed by using the Arima pipeline (https://github.com/ArimaGenomics/mapping_pipeline), which involves processing and mapping of Hi-C reads with samtools v.1.9 (Danecek et al. 2021) and bwa v.0.7.17-r1188 (Li and Durbin 2009), respectively, and deduplication with Picard v.2.27.5 (http://broadinstitute.github.io/picard/). The final scaffolding step was performed with YaHS v.1.2 (Zhou et al. 2023), followed by a manual processing step with JuicerTools v.2.20.00 (https://github.com/aidenlab/JuicerTools), gap closing with TGS-GapCloser v.1.2.1 (Xu et al. 2020), and a manual curation step with Juicebox desktop v.2.17.00 (https://aidenlab.gitbook.io/juicebox/desktop). The resulting assembly was used for a second round of Hi-C scaffolding and gap closing to obtain the final genome assembly. Mitochondrial contigs were identified by performing BLAST v.2.13.0 (Altschul et al. 1990) searches of the contigs against the *Z*. *viviparus* mitochondrial genome (with -max_target_seqs 100 -max_hsps 100 -evalue 1e−25) and removed from the nuclear genome assembly.

Repeat families for the three fish species were independently identified and classified with RepeatModeler2 v2.0.5 (Flynn et al. 2020) and resources from Dfam (Storer et al. 2021). After combining the three repeat libraries, redundancy was reduced and false positives were omitted using MCHelper (Orozco-Arias et al. 2023). Repeats were then masked in all genomes with the combined repeat library using RepeatMasker v4.1.8 (Smit et al. 2013–2015, http://repeatmasker.org) and helper scripts from TETools v1.88 (https://github.com/Dfam-consortium/TETools) to create a soft-masked genome assembly. Tandem repeats were identified with Phobos v3.3.12 (Mayer 2007; Mayer et al. 2010) for a unit size range of 1 to 50 bp, and the telomeric repeat motif (TTAGGG/CCCTAA)*_n_* (Moyzis et al. 1988) was searched at the beginning/end of the 24 main scaffolds to ascertain whether the telomeric regions were successfully assembled (supplementary tables S1–S3, Supplementary Material online; for details, see supplementary section S3, Supplementary material online).

### RNA Extraction and Sequencing

For transcriptomic long-read sequencing, RNA was extracted from muscle (∼25 mg) and liver (∼20 mg) tissues using the RNeasy Plus Mini kit (Qiagen). A TapeStation 2200 and the RNA ScreenTape Analysis kit (both Agilent) were used for RNA quantification and quality assessment. Further sample processing and sequencing were performed at the WGGC. Full-length cDNA sequences were produced using the TeloPrime Full-Length cDNA Amplification kit V2 (Lexogen). SMRTbell adapters were added using the SMRTbell prep kit 3.0 (PacBio), followed by RNA-seq on PacBio Sequel II systems.

These transcriptomes were complemented with Illumina short-read data generated from 12 Swedish specimens, caught in May 2023 in the Gullmarn Fjord, Lysekil, Sweden (location: latitude 58.23, longitude 11.41). Total RNA was extracted from 5 and 10 mg of liver tissue using the RNeasy Plus Micro and RNeasy Plus Mini kits (Qiagen), respectively. RNA concentrations were quantified with a Quantus fluorometer and the QuantiFluor RNA System kit (both Promega). The sample quality was checked on a Fragment Analyzer with the RNA kit (15 nt) (both Agilent). Library preparation and sequencing were performed at the WGGC. The VAHTS Universal V8 kit (Vazyme) was used for poly-A selection of mRNA from 100 ng of total RNA and cDNA library construction. The 12 libraries were paired-end sequenced (100 bp) on an Illumina NextSeq2000.

### Gene Prediction

We employed a customized version of the BRAKER3 v3.0.4 (Hoff et al. 2016; Brůna et al. 2021; Gabriel et al. 2024) protocol to integrate evidence from short- and long-read transcriptomic data as well as from protein data of the vertebrate OrthoDB partition (Kuznetsov et al. 2023).

First, the Illumina RNA-seq libraries were used for gene prediction. Homopolymers at the end of the reads were removed with a custom C++ program, followed by quality trimming using the cutadapt v3.4 (Martin 2011) wrapper script TrimGalore! v0.6.10 (Krueger 2024, https://github.com/FelixKrueger/TrimGalore) in paired-end mode, retaining only reads with a minimum length of 25 bp. HISAT2 v2.1.0 (Kim et al. 2019) was used to align the trimmed reads against the soft-masked genome assembly in strand-specific mode. The resulting .bam files were sorted with samtools and used to run GeneMark-ETP (Bruna et al. 2024).

A second gene set was predicted based on evidence gained from PacBio Iso-Seq reads, which were splice-aware aligned against the soft-masked genome using minimap2 v2.26 (Li 2018), followed by .bam file sorting with samtools. A singularity container specifically set up for long-reads (Hoff, https://hub.docker.com/r/katharinahoff/playground) was used to run GeneMark-ETP.

The final structural genome annotation was created by combining both gene set predictions using TSEBRA v1.1.2 (Gabriel et al. 2021). No functional annotation was performed.

### Assembly Evaluation and Comparative Analyses

Assembly statistics were calculated using the bbmap v38.82 (Bushnell 2014) script stats.sh and QUAST v.5.2.0 (Gurevich et al. 2013). K-mer distributions in the Hifi reads as well as in the haplotype resolved assembly before and after scaffolding (supplementary fig. S3, Supplementary Material online) were estimated with Merqury v.1.3 (Rhie et al. 2020). The final assembly was screened for contamination with BlobToolKit v.4.3.3 (Challis et al. 2020).

As no randomly generated Illumina data were available, a genome size estimation based on k-mer frequencies was not possible. Therefore, we estimated the genome size (*G*) from the PacBio HiFi reads as


G=B/Cov

where *B* refers to the cumulative length sum from all HiFi reads incorporated in the assembly and *Cov* to the average genome coverage of HiFi reads. Both parameters were obtained with bash shell commands and samtools.

To evaluate the assembly completeness, the BUSCO program v.5.4.6 and database were used to search for BUSCO genes defined for ray-finned fish (Actinopterygii_odb10) in the genome (-m genome) and in the predicted protein set (-m transcriptome *or* -m protein).

Genome comparisons were made between *Z*. *viviparus* and its closest relatives with chromosome-level genome assemblies i.e. *M*. *gelatinosum* (GenBank GCA_949748355.1) and *L*. *pacificus* (GenBank GCA_028022725.1). Pairwise genome alignments were conducted with minimap2, and assembly consistency plots were produced with the Circos (Krzywinski et al. 2009) wrapper script Jupiter (Chu 2024, https://github.com/JustinChu/JupiterPlot).

## Supplementary Material

evae155_Supplementary_Data

## Data Availability

All data are available at the NCBI under Bioproject PRJNA1068064. Raw sequencing data SRA accession numbers: SRR27896878 (PacBio HiFi reads), SRR27896879 (Illumina Hi-C reads), SRR28471744-SRR28471745 (PacBio Iso-Seq reads), and SRR27885821-SRR27885832 (Illumina RNA-seq reads). Genome accession numbers: The annotated genome assembly is deposited under GCA_040110945.1 at GenBank and the Whole Genome Shotgun project is deposited at DDBJ/ENA/GenBank under the master accession JBCEZU000000000. Mitogenome accession number: The mitochondrial genome is deposited at GenBank under PP556339.
