# Supplementary material for: Chromosome-Level Genome Assembly of the Viviparous Eelpout Zoarces viviparus
Source: Genome Biol Evol. 2024 Jul 17;16(8):evae155. doi: 10.1093/gbe/evae155 (PMC11331339; doi:10.1093/gbe/evae155)
Supplement: evae155_Supplementary_Data [file evae155_supplementary_data.pdf]

Supplementary information for

Chromosome-level genome assembly of the viviparous eelpout *Zoarces viviparus*

Nico Fuhrmann[1,#], Marie V. Brasseur[1,#,*], Christina E. Bakowski[2], Lars Podsiadlowski[2], Stefan Prost[3,4,5,+], Henrik Krehenwinkel[1,+], Christoph Mayer[2,+]

[#] These authors contributed equally and share first authorship.

[+] These authors contributed equally and share last authorship.

Name	OrcID	E-mail
Nico Fuhrmann	0000-0001-6613-2821	fuhrmannn@uni-trier.de
Marie Brasseur	0000-0003-2044-4867	m.brasseur@leibniz-lib.de
Christina Bakowski	0009-0009-6938-6195	s6chbako@uni-bonn.de
Lars Podsiadlowski	0000-0001-7786-8930	l.podsiadlowski@leibniz-lib.de
Stefan Prost	0000-0002-6229-3596	stefan.prost@oulu.fi
Henrik Krehenwinkel	0000-0001-5069-8601	krehenwinkel@uni-trier.de
Christoph Mayer	0000-0001-5104-6621	c.mayer@leibniz-lib.de

Affiliations

[1]Department of Biogeography, Trier University, Trier 54296, Germany

[2]Leibniz Institute for the Analysis of Biodiversity Change (LIB), Bonn 53113, Germany

[3]Ecology and Genetics Research Unit, University of Oulu, Oulu 90014, Finland

[4]South African National Biodiversity Institute, National Zoological Garden, Pretoria 0002, South Africa.

[5]Central Research Laboratories, Natural History Museum Vienna, Vienna 1010, Austria

***Author for Correspondence:** Marie V. Brasseur, Department of Biogeography, Trier University, Trier, Germany, m.brasseur@leibniz-lib.de

Section S1: Nuclear genome characteristics

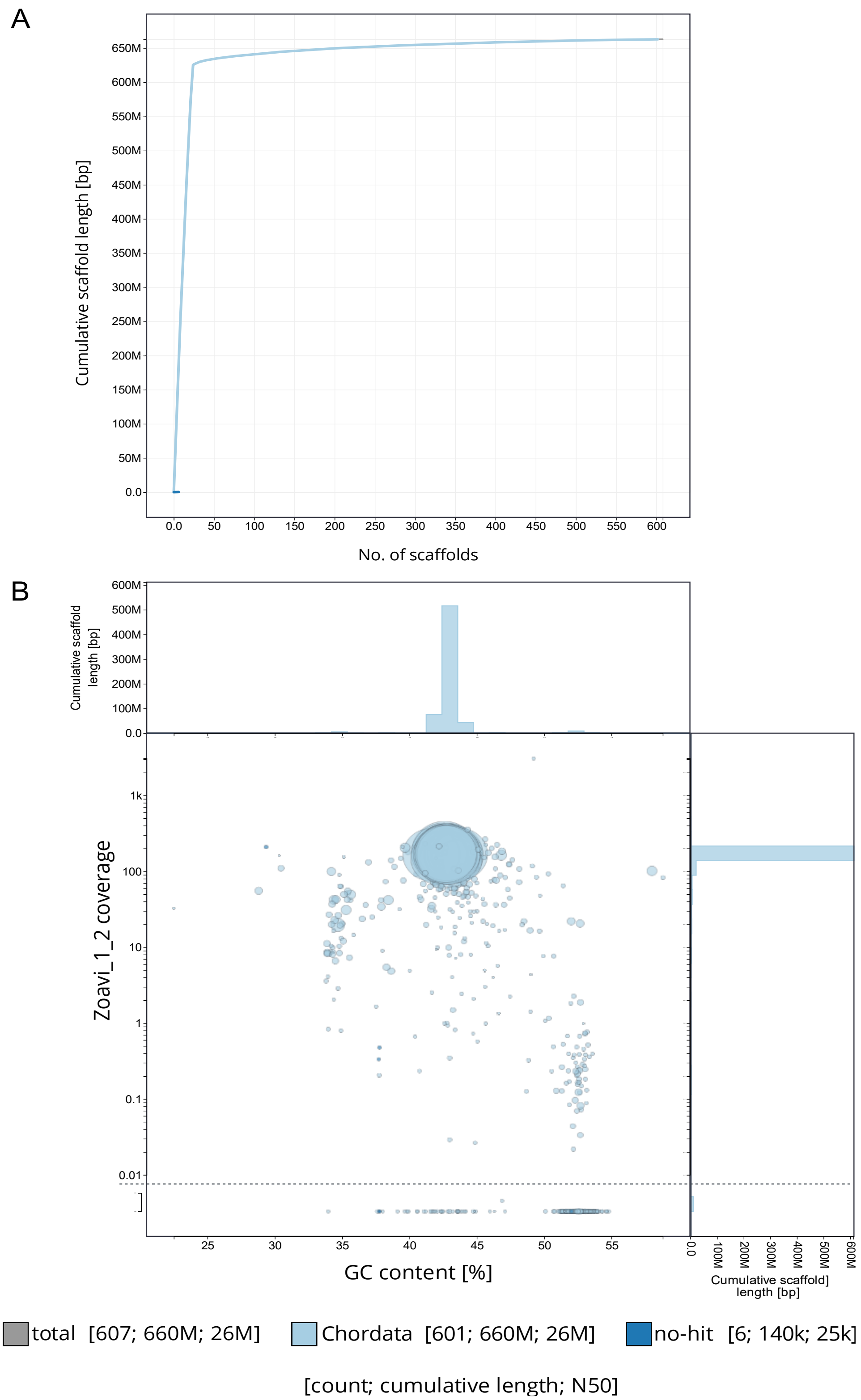

Fig. S1: BlobPlot analysis, depicting the relation between (A) the cumulative length of the scaffolds versus the cumulative base count, showing that 94% of the genome are assembled in the first 24 scaffolds, which correspond to the 24 chromosomes of the haploid *Z. viviparus* genome, and (B) the logarithmic genome coverage (y-axis) against the GC content (x-axis). The taxonomic assignment of contigs indicates that no contamination is present.

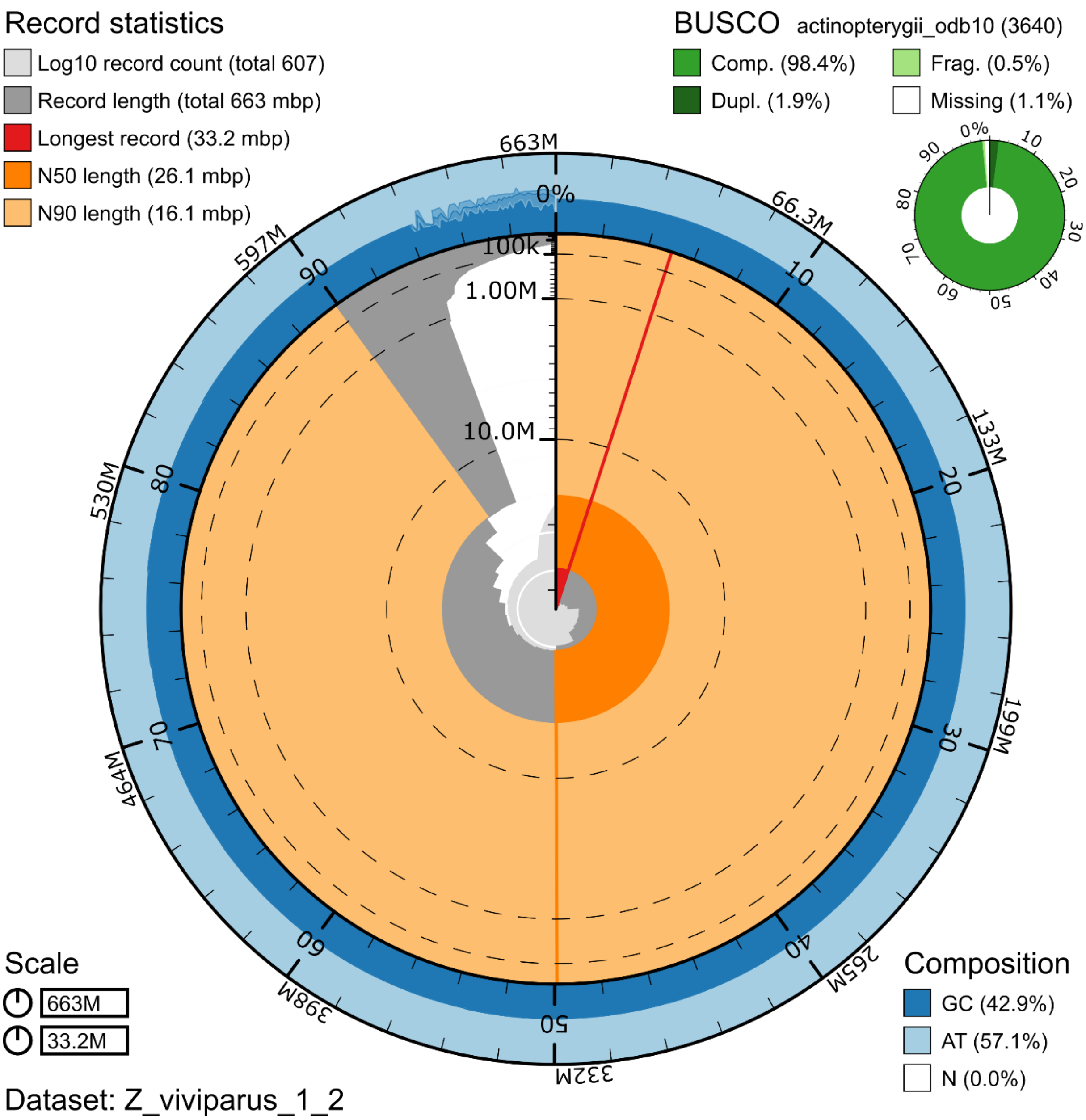

Fig. S2: Snail plot presenting statistics of the *Z. viviparus* genome assembly (Z_viviparus_1_2). The plot is organised in bins, each comprising 2% of the 663 mbp assembly. The dark and light blue arcs represent the GC/AT content across the genome whereas the dark and light orange arcs indicate the scaffolds used to calculate the N50 and N90 lengths on the radial scale, respectively. The scaffold length distribution is shown in dark grey on a logarithmic radial scale where dashed lines show successive orders of magnitude. The longest scaffold is shown in red (chromosome 1, 33.2 mbp). The light grey spiral displays the cumulative scaffold length on the decadic logarithmic scale, whereby the white scale lines show successive orders of magnitude.

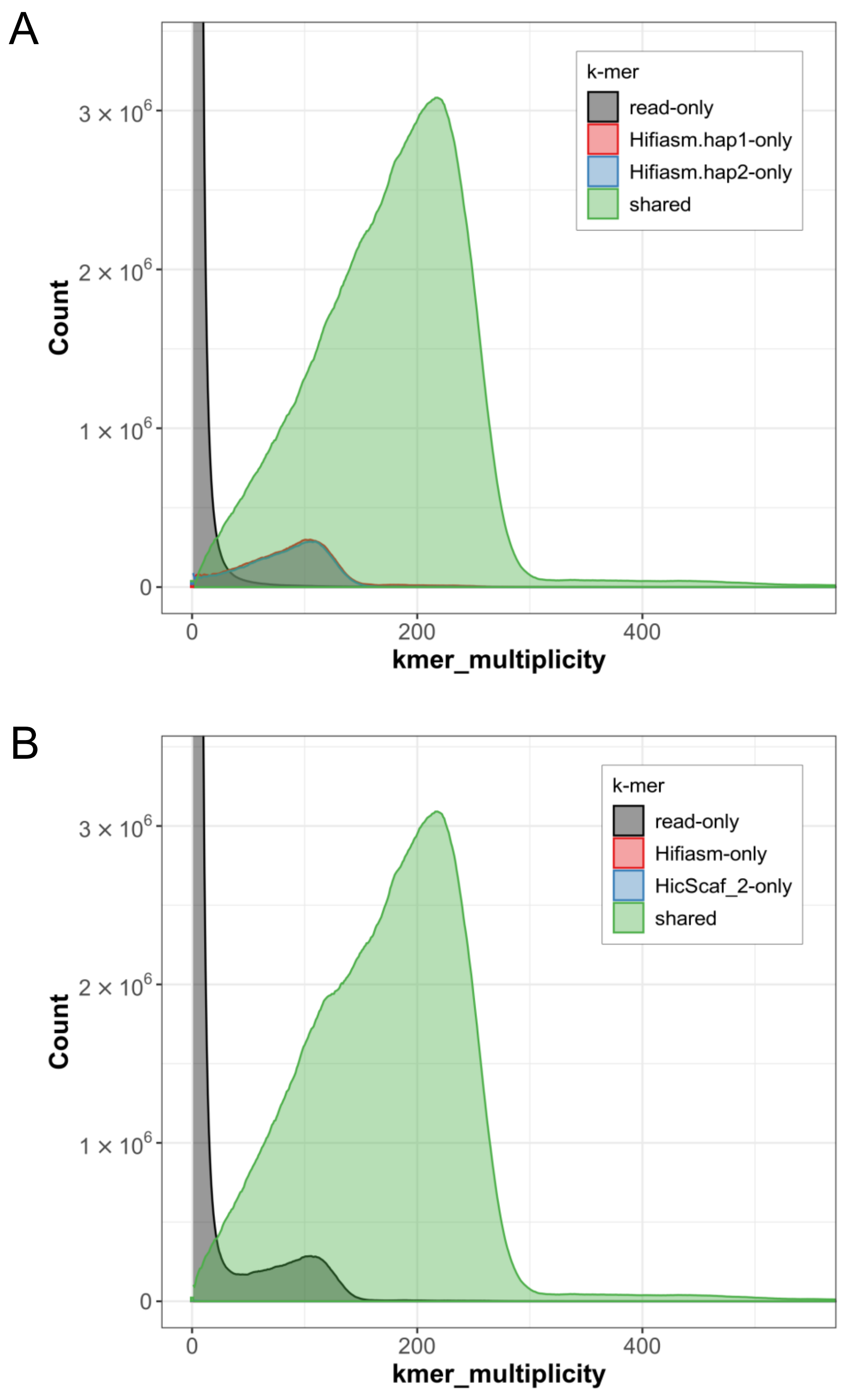

Fig. S3: Merqury assembly spectrum plots between (A) the haplotypes initially generated by Hifiasm and (B) the haplotype resolved final assembly i.e., after the scaffolding and gap closing procedure. The high proportion of shared k-mers (green) indicate that no irregularities were introduced due to technical artefacts (e.g., bias during sequencing and/or assembly).

Section S2: Mitochondrial genome

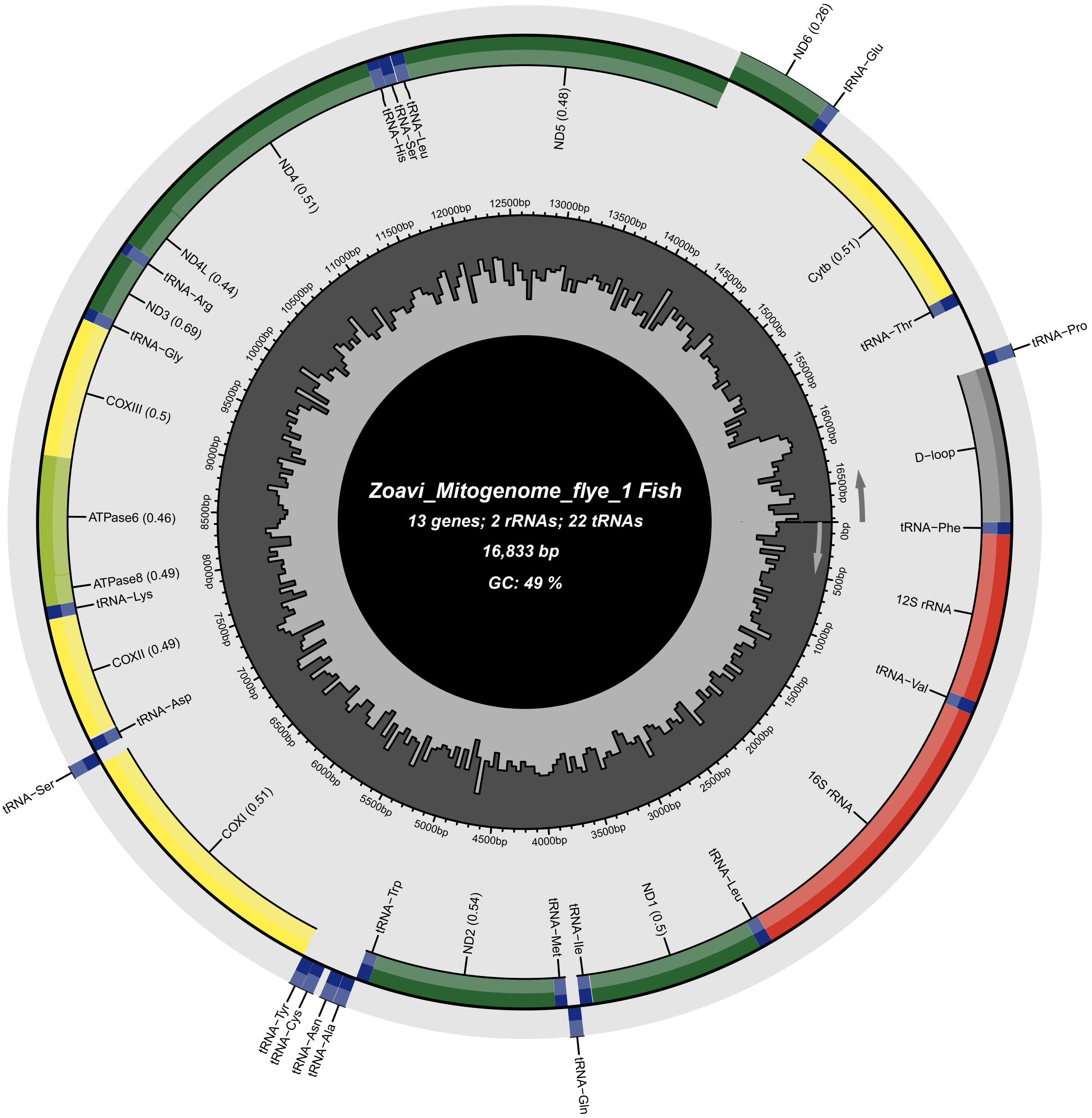

Fig. S4: The assembled and annotated mitochondrial genome of *Z. viviparus*.

Section S3: Tandem repeat analyses

First, we analysed tandem repeat (TR) patterns in the *Z. viviparus* genome and in the genome of close relatives: two other eelpouts belonging to the same family (Zoarcidae) i.e., *Melanostigma gelatinosum* (NCBI accession GCA_949748355.1) and *Lycodes pacificus* (NCBI accession GCA_028022725.1) and further, three species belonging to the same infraorder (Zoarcales), i.e. *Leptoclinus maculatus* (NCBI accession GCA_032191485.1)*, Anarrhichthys ocellatus* (NCBI accession GCA_004355925.1) and *Pholis gunnellus* (NCBI accession GCA_910591455.2).

Imperfect TRs were identified with the Phobos software v3.3.12 using the command line parameters "-s 10 -U 51 --outputFormat 1 --NsAsMissense". This identifies TRs in the unit size range 1-51 bp. Since the highest unit length can include imperfect matches to TRs of even longer unit lengths, we used the resulting data to only analyse the repeat content in the size range 1-50 bp. A TR is reported if it has a minimum score of 10, whereby each match gets a score of +1 and each mismatch or gap a score of -5. The first unit is not scored. Unknown nucleotides are treated as mismatches. Repeat patterns reported below are always reported as normalised form. A normalised repeat pattern represents all patterns that differ from the normalised form by (i) cyclic permutations of the repeat pattern and (ii) cyclic permutations of the reverse complement. The normalised form is the pattern that comes first when sorting all patterns it represents alphabetically.

TRs found in the assemblies of the *Z. viviparus* genome and genomes of relatives

The proportion of nucleotides in the genome assembly of *Z. viviparus* that belong to TRs is 7%, which is comparable to genome assemblies of other species belonging to the infraorder Zoarcales (Fig. S5). The highest repeat content was found in the genome assembly of *M. gelatinosum* with a genomic coverage > 10%, with a particularly high coverage contributed by TRs in the length range 11-50 bp.

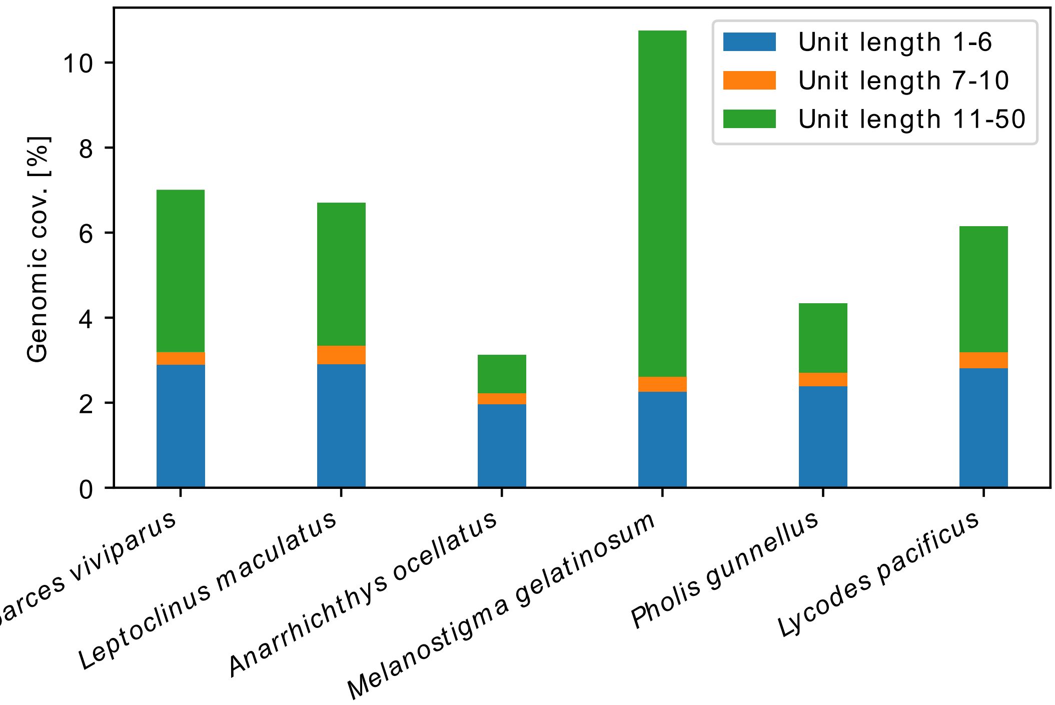

Fig. S5: Comparison of genomic TR coverage for the genome assemblies of *Z. viviparus, L. maculatus, A. ocellatus, M. gelatinosum, P. gunnellus* and *L. pacificus* for TRs in different repeat unit length classes.

In most of the analysed genomes, short tandem repeats with a unit size of 1-6 bp are dominant (Fig. S6). One notable exception is the *M. gelatinosum* genome, in which we detected a single dominant repeat pattern i.e., AAAAAAAAATATTTTTTTAGTTACTTTGG, with a genomic coverage of 4.1%. In *Z. viviparus*, di-nucleotide repeats have the highest genomic coverage (1.8%), followed by 28 bp repeats (1.1%). and 20 bp repeats (0.3%).

Z. viviparus has 29 repeats that are more than 100,000 bp long (Table S1). Notably, all long repeats are based on only a few repeat patterns. The longest connected repeat array was found on chromosome 18 and is 1,831,213 bp long, starting at nucleotide 4 and stretches until nucleotide 1,831,216. This repeat was split by Phobos into three overlapping parts. The first "split" is due to a shift in the alignment. The second split results from a shift in the pattern (Table S1). The repeat has a pattern length of 41 bp in the first two parts and a pattern length of 20 bp in the third part of the repeat (Table S1). After this 1.8 mega base pairs repeat, we found 96 bp before the same 20 bp repeat pattern is found again for another 158,801 bp. So effectively, chromosome 18 starts with a 1,990,110 bp long repeat array. The main contributor to the 28 bp peak found for the *Z. viviparus* genome (Fig. S6) is a 28 bp pattern

(AATGAGTGAGGTCATGTCAGACCAGTGC) which only occurs in 35 repeat loci, but has a total genomic coverage of 0.7%. The longest repeat with this pattern is found in chromosome 8 in the coordinate range 25,904,445 to 26,592,201 with a repeat length of 687,757 bp and a perfection of 92.7%. Patterns that differ only by a single nucleotide substitution are found as the main pattern in several long TRs (Table S1). Moreover, several large unplaced scaffolds consist entirely of a single repeat with this pattern or one of its almost identical variants (Table S2). None of these long repeats were softmasked by RepeatMasker.

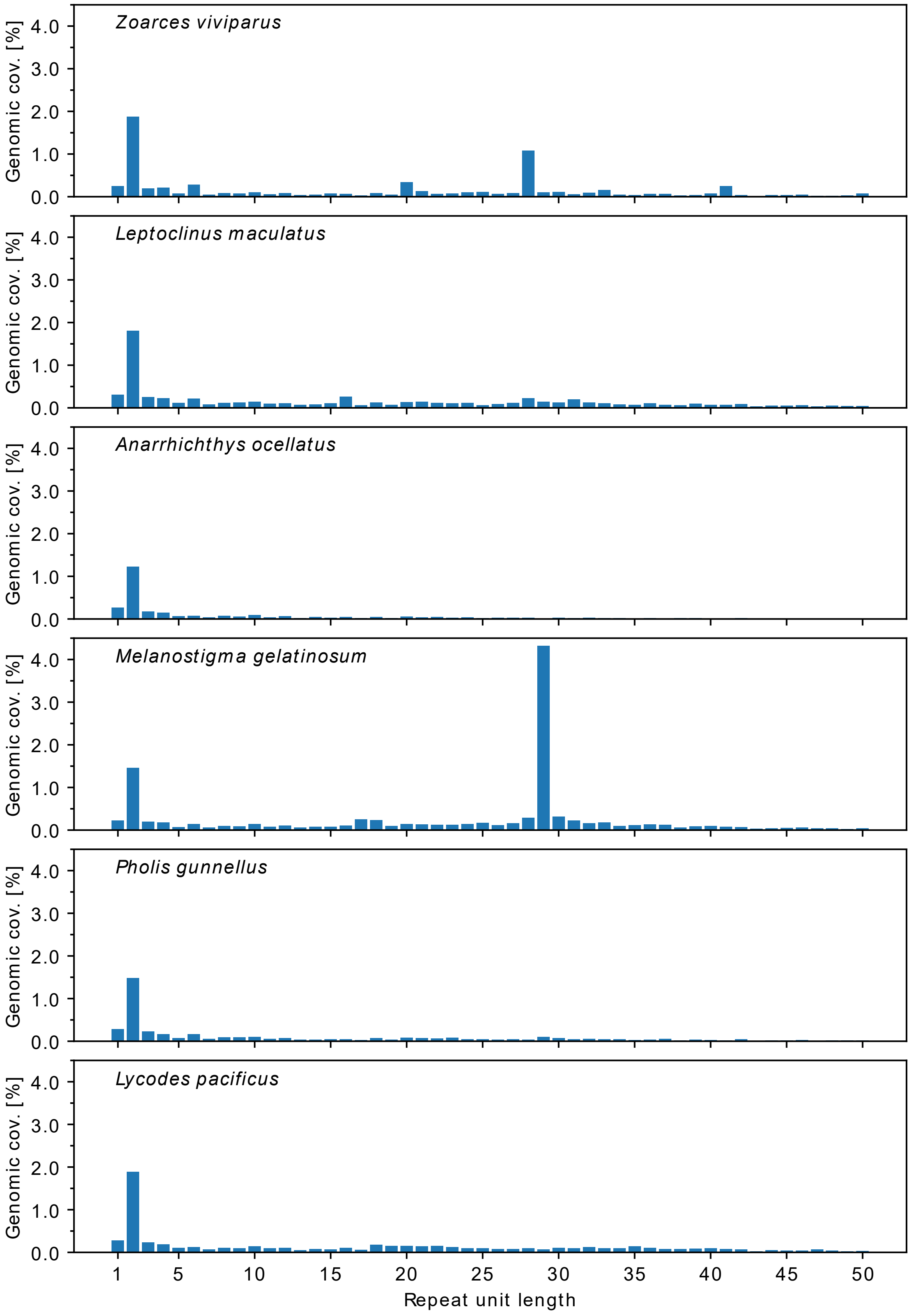

Fig. S6: Comparison of genomic TR coverage for individual repeat unit lengths.

Table S1: *Z. viviparus* has 29 repeats in the unit size range 1-50 with a length longer than 100,000 bp. The two repeats marked by (*) are overlapping and have the same repeat pattern. They are reported as two separate repeats, since Phobos could not align the repeat across a larger gap in the alignment. All longer repeats across multiple chromosomes are based on a small number of repeat patterns, indicated by colours in the table. Similar repeat patterns have similar colours. Some repeat units consist of multiple similar copies themself.

Scaffold	Unit length	Start coord.	End coord.	Length	Length in alignment	Score	Perfection [%]	Repeat Unit
Chromosome 2	28	31151036	31258440	107405	107505	57130	92.144	AATGAGTGAGGTCATGTCAGACCAG TGC
Chromosome 4	45	24045493	24175195	129703	129841	42686	88.658	AATATGA*C*CTCATAGAATATGA*T*CTC ATAGAATATGA*T*CTCATAG
Chromosome 5	28	28296438	28463012	166575	166595	81998	91.463	AATGAGTGAGGTCATGTCAGACCAG TGC
Chromosome 6	33	2685500	2793957	108458	105892	5064	82.874	AAACTCCTGAGGGAATGGGAATAAA GAGGAAAG
Chromosome 7	28	23166023	23308496	142474	142439	72585	91.772	AATGAGTGA*T*GTCATGTCAGACCAG TGC
Chromosome 8	28	25904445	26592201	687757	687839	390090	92.735	AATGAGTGAGGTCATGTCAGACCAG TGC
Chromosome 8	28	27115583	27722575	606993	606890	367746	93.390	AATGAGTGAGGTCATGTCAGACCAG TGC
Chromosome 8	28	29119604	29326779	207176	207189	121468	93.075	AATGAGTGAGGTCATGTCAGACCAG TGC
Chromosome 8	20	29410031	29646122	236092	236163	110585	91.115	AAAAAAGATGCGAACATTCT
Chromosome 9	28	348461	710605	362145	361961	159058	90.547	AATGAGTGAGGTCATGTCAGACCAG TGC
Chromosome 10	28	26709020	26866605	157586	157618	75001	91.231	AATGAGTGAGGTCATGTCAGACCAG TGC
Chromosome 12	28	24657414	24798660	141247	141017	65413	90.976	AAACTAATACGAATACAATAAGTGC AAC
Chromosome 14	28	24140447	24490785	350339	350129	115673	88.672	AATGAGTGAGGTCATGTCAGACCAG TGC
Chromosome 16	28	25945847	26100452	154606	154564	84155	92.337	AATGAGTGAGGTCATGTCAGACCAG TGC
Chromosome 18 (*)	41	4	603273	603270	617771	245454	89.912	AAAAAAGATGCGAACATTCT AAAAAAGATTGCGAACATTCT

Chromosome 18 (*)	41	603271	1094838	491568	503838	225318	90.769	AAAAAAGATGCGAACATTCT AAAAAAGATTGCGAACATTCT
Chromosome 18 (*)	20	1094837	1831216	736380	736429	336802	90.906	AAAAAAGATGCGAACATTCT
Chromosome 18	20	1831310	1990110	158801	158895	83805	92.096	AAAAAAGATTCGAACATTCT
Chromosome 18	28	2570289	2939440	369152	368996	171988	90.971	AATGAGTGAGGTCATGTCAGACCAGTGC
Chromosome 20	33	21211629	21337596	125968	125842	67689	92.191	AAAATGCTGTTACAATCCGTTAACCTGTAGTTT
Chromosome 21	28	26200160	26628422	428263	427988	203826	91.176	AATGAGTGA*T*GTCATGTCAGACCAGTGC
Chromosome 24	28	415289	542064	126776	126716	74634	93.089	AATGAGTGAGGTCATGTCAGACCAGTGC
Scaffold 26	28	181819	581213	399395	399136	190058	91.167	AATGAGTGAGGTCATGTCAGACCAGTGC
Scaffold 33	40	113544	370999	257456	257476	117373	90.868	AAAAAAGATGCGAACATTCT AAAAAAGATTCGAACATTCT
Scaffold 38	41	2088	263658	261571	268082	147481	92.488	AAAAAACGATGCGAACATTCT AAAAAAGATGCGAACATTCT
Scaffold 47	28	5	197000	196996	196975	99885	91.717	AATGAGTGAGGTCATGTCAGACCACTGC
Scaffold 55	28	1	174222	174222	174241	101753	93.033	AATGAGTGAGGTCATGTCAGACCAGTGC
Scaffold 63	28	20189	148998	128810	128888	76792	93.240	AATGAGTGA*T*GTCATGTCAGACCAGTGC
Scaffold 70	28	5	139996	139992	139991	74446	92.159	AATGAGTGAGGTCATGTCAGACCAGTGC

Table S2: Scaffolds consisting entirely of variants of a single repeat pattern. TRs are coloured consistently with Table S1.

Scaffold	Scaffold length	Scaffold coverage	Pattern	Repeat perfection
Scaffold 38	269562	99.23%	AAAAAACGATGCGAACATTCT<br>AAAAAAGATGCGAACATTCT	92.5%
Scaffold 47	197000	100%	AATGAGTGAGGTCATGTCAGACCACTGC	91.7%
Scaffold 55	174225	100%	AATGAGTGAGGTCATGTCAGACCAGTGC	93.0%
Scaffold 70	140000	100%	AATGAGTGAGGTCATGTCAGACCAGTGC	92.2%
Scaffold 147	85000	100%	AATGAGTGAGGTCATGTCAGACCAGTGC	94.6%
Scaffold 158	80000	100%	AATGAGTGAGGTCATGTCAGACCAGTGC	94.3%
Scaffold 170	75000	99.99%	AATGAGTGAGGTCATGTCAGACCAGTGC	94.6%
Scaffold 404	32737	99.99%	AAAAAAGATGCGAACATTCT	91.6%
Scaffold 498	25000	100%	AATGAGTGATGTCATGTCAGACCAGTGC	92.3%

Telomeric repeat analysis

To further explore the chromosome-level nature of our *Z. viviparus* genome assembly, we searched for the telomeric repeat motif $(TTAGGGx/CCCTAAx)_n$ using a C++ program written by C. Mayer, whereby *x* can represent one or more additional bases. This TR has been first described in in the telomeric regions of human chromosomes (Moyzis et al. 1988), but was shown to be conserved across vertebrates including fish (Ocalewicz 2013) and is even considered the ancestral telomere repeat motif of metazoans (Traut et al. 2007). To identify telomeric regions within the *Z. viviparus* genome, we searched for exact matches of TTAGGG/CCCTAA in the first (5' end) and last (3' end) 40 kilo base pairs of the 24 main scaffolds of the assembly (Tab. S3). While the TR was clearly present at the beginning and/or end of eight scaffolds, no significant accumulation of the TR was found in the remaining 16 scaffolds. In these 16 scaffolds, a variant of the TR motif might be present, or the telomeres were not successfully assembled. In fact, the assembly of repetitive sequence elements such as

telomeres is challenging. For the *Z. viviparus* genome assembly, this problem is likely aggravated by its high repeat content, especially observed in the 16 scaffolds in which we could not detect the standard vertebrate telomere repeat motif (Tables S1 - S3).

Table S3: Telomeric repeat content in the 24 main scaffolds of the *Z. viviparus* genome assembly. Repetitions > 100 of the TTAGGG motif and its reverse complement CCCTAA are indicated in bold.

Scaffold	5' end (TTAGGG)	3'end (TTAGGG)	5'end (CCCTAA)	3'end (CCCTAA)
Chromosome 1	12	5	0	5
Chromosome 2	0	4	0	10
Chromosome 3	1	1	1	2
Chromosome 4	1	6	2	5
Chromosome 5	7	0	4	0
Chromosome 6	7	3	7	3
Chromosome 7	2	1	**2329**	3
Chromosome 8	7	1	9	3
Chromosome 9	12	5	**1193**	**112**
Chromosome 10	0	3	12	0
Chromosome 11	0	**514**	**1518**	4
Chromosome 12	1	1	**3107**	2
Chromosome 13	5	6	3	4
Chromosome 14	1	0	1	0
Chromosome 15	1	7	**1196**	5
Chromosome 16	3	0	5	0
Chromosome 17	9	3	**1397**	1
Chromosome 18	0	0	0	0
Chromosome 19	6	1	5	**247**
Chromosome 20	8	0	7	0
Chromosome 21	11	0	4	0
Chromosome 22	4	1	**3450**	5
Chromosome 23	5	3	3	5
Chromosome 24	2	8	6	0

Bibliography

Moyzis RK et al. 1988. A highly conserved repetitive DNA sequence, (TTAGGG)n, present at the telomeres of human chromosomes. Proc. Natl. Acad. Sci. 85:6622–6626. doi: 10.1073/pnas.85.18.6622.

Ocalewicz K. 2013. Telomeres in Fishes. Cytogenet. Genome Res. 141:114–125. doi: 10.1159/000354278.

Traut W et al. 2007. The telomere repeat motif of basal Metazoa. Chromosome Res. doi: 10.1007/s10577-007-1132-3.
